# Evidence of Face Masks and Masking Policies for the Prevention of SARS-CoV-2 Transmission and COVID-19 in Real-World Settings: A Systematic Literature Review

**DOI:** 10.3390/ijerph22101590

**Published:** 2025-10-20

**Authors:** Noe C. Crespo, Savannah Shifflett, Kayla Kosta, Joelle M. Fornasier, Patricia Dionicio, Eric T. Hyde, Job G. Godino, Christian B. Ramers, John P. Elder, Corinne McDaniels-Davidson

**Affiliations:** 1 School of Public Health, San Diego State University, San Diego, CA 92123, USA; sshifflett@sdsu.edu (S.S.); kostakayla@gmail.com (K.K.); jfornasier7548@sdsu.edu (J.M.F.); pdionicio8044@sdsu.edu (P.D.); ehyde@health.ucsd.edu (E.T.H.); jelder@sdsu.edu (J.P.E.); cmcdaniels@sdsu.edu (C.M.-D.); 2 Institute for Behavioral and Community Health (IBACH), San Diego, CA 92123, USA; 3 Laura Rodriguez Research Institute, Family Health Centers of San Diego, San Diego, CA 92101, USA; jobg@fhcsd.org (J.G.G.); christianr@fhcsd.org (C.B.R.)

**Keywords:** SARS-CoV-2, masks, real-world settings

## Abstract

**Objectives:** Prevention of severe acute respiratory syndrome coronavirus 2 (SARS-CoV-2) and the disease COVID-19 is a public health priority. The efficacy of non-pharmaceutical interventions such as wearing face masks to prevent SARS-CoV-2 infection has been well established in controlled settings. However, evidence for the effectiveness of face masks in preventing SARS-CoV-2 transmission within real-world settings is limited and mixed. The present systematic review evaluated the effectiveness of face mask policies and mask wearing to prevent SARS-CoV-2 transmission and COVID-19 in real-world settings. **Methods:** Following PRISMA guidelines, scientific databases, and gray literature, were searched through June 2023. Inclusion criteria were as follows: (1) studies/reports written in or translated to English; (2) prospectively assessed incidence of SARS-CoV-2 or COVID-19; (3) assessed the behavior and/or policy of mask-wearing; and (4) conducted in community/public settings (i.e., not laboratory). Studies were excluded if they did not parse out data specific to the effect of mask wearing (behavior and/or policy) and subsequent SARS-CoV-2 transmission or COVID-19 disease or if they relied solely on statistical models to estimate the effects of mask wearing on transmission. A total of 2616 studies were initially identified, and 470 met inclusion and exclusion criteria for full-text review. The vote counting method was used to evaluate effectiveness, and risk of bias was assessed using JBI critical appraisal tools. **Results:** A total of 79 unique studies met the final inclusion criteria, and their data were abstracted and evaluated. Study settings included community/neighborhood settings (n = 34, 43%), healthcare settings (n = 30, 38%), and school/universities (n = 15, 19%). A majority of studies (n = 61, 77%) provided evidence to support the effectiveness of wearing face masks and/or face mask policies to reduce the transmission of SARS-CoV-2 and/or prevention of COVID-19. Effectiveness of mask wearing did not vary substantially by study design (67–100%), type of mask (77–100%), or setting (80–91%), while 85% of masking policies specifically reported a benefit. **Conclusions:** This systematic literature review supports public health recommendations and policies that encourage the public to wear face masks to reduce the risk of SARS-CoV-2 infection and COVID-19 in multiple real-world settings. Effective communication strategies are needed to encourage and support the use of face masks by the general public, particularly during peak infection cycles.

## 1. Introduction

Since the World Health Organization (WHO) declared COVID-19 a pandemic in March of 2020 [1], the U.S. and other countries have experienced several waves of respiratory syndrome coronavirus 2 (SARS-CoV-2) infections, which put great strain on hospitals and the healthcare system [2]. SARS-CoV-2 is responsible for the disease COVID-19, and in 2020, COVID-19 was the third leading cause of death in the U.S. and the number one cause of death for people aged 45–84 years [3]. As of June 2023, there have been over 6.9 million reported deaths globally due to COVID-19, and minoritized/underserved populations have been disproportionately affected [3].

Public health recommendations for the prevention of SARS-CoV-2 infection include vaccination, masking, social distancing, avoiding crowds and poorly ventilated spaces, frequent washing of hands, and disinfecting high touch surfaces [4]. Nonpharmaceutical interventions (NPIs), which are actions that persons and communities can take to help slow the spread of respiratory virus infections, are often the most readily available interventions to help slow the transmission of infectious viruses in communities [5]. Wearing personal protective equipment (PPE), such as face masks, has long been used in previous pandemics (e.g., 1920 influenza), epidemics, and outbreaks as an effective strategy to protect against the spread of respiratory infectious disease [6]. Despite ample evidence for the efficacy and effectiveness of PPE to prevent the transmission of respiratory infections (e.g., seasonal influenza, tuberculosis, etc.), the adoption of these preventive measures by the general public has varied widely. The emergence of increasingly infectious variants such as Delta and Omicron renewed questions about the role of masks to prevent the spread of SARS-CoV-2 [7]. These variants contain mutations that affect transmissibility and virulence, potentially impacting the efficacy and effectiveness of vaccines, therefore increasing the need to enact NPIs to help contain viral spread, particularly during peak outbreaks [8].

Enacting policies that require people to use face masks in public to curb the spread of SARS-CoV-2 has been a politicized issue [9]. Misinformation surrounding the efficacy and effectiveness of face masks can threaten the health of the public. At the height of the COVID-19 pandemic, a subset of the U.S. population expressed strong opposition to wearing masks and mask mandates through social media channels (e.g., Twitter) [10]. Some of the most common reasons cited in opposition to wearing masks were the belief that wearing masks was not necessary for certain groups (e.g., children and healthy individuals), mask mandates infringed upon personal liberty, and that masks were uncomfortable and not effective at preventing SARS-CoV-2 infection [10]. Thus, policymakers and public health officials need better quality evidence for the effectiveness of masking policies and the use of masks to provide evidence-based guidance to the public [11].

Previous literature reviews show that masks and facial coverings are efficacious at reducing the risk of SARS-CoV-2 transmission in controlled laboratory settings [11,12,13]; however, the effectiveness of masks in real-world settings (e.g., schools, community settings, transportation, and hospitals) have not been well documented. The effectiveness of masks in real-world settings are impacted by multiple factors including the level of adherence, the mask type, and other environmental contexts [14]. Overall, findings regarding the effectiveness of mask wearing in community settings remain limited to date. Leech et al. [15] used a Bayesian hierarchical model approach across 92 regions and found that self-reported mask wearing was associated with a 25% reduction in SARS-CoV-2 transmission, although mask mandates themselves were not associated with transmission rates. In contrast, Bundgaard et al. [16] reported no significant difference in SARS-CoV-2 infection rates between participants who wore masks and those who did not. One of the earliest systematic reviews in community settings provided promising preliminary evidence for the effectiveness of mask wearing; however, the evidence was limited due to the small number of studies available at that time [17]. These mixed results highlight the need for an updated and synthesized review of the effectiveness of mask policies and wearing face masks in non-controlled, real-world settings. The purpose of this review was to evaluate the effectiveness of mask-wearing and masking policies for the prevention of SARS-CoV-2 transmission and COVID-19 in multiple real-world settings.

## 2. Methods

### 2.1. Data Sources

A systematic search of electronic databases was conducted in SCOPUS, PubMed, and CINAHL. Gray literature, such as conference abstracts, county data, and government reports, was also identified through ProQuest, MedRxiv, BioRxiv, and Google Scholar. Key terms were used to capture the relevant literature on making policies, mask wearing, and SARS-CoV-2 transmission and COVID-19. Table 1 summarizes the key terms used for this review. The study protocol was registered in the International Platform of Registered Systematic Review and Meta-analysis Protocols (INPLASY202460011).

### 2.2. Study Selection

Study eligibility criteria were as follows: (1) written in or translated to English; (2) prospectively assessed incidence of SARS-CoV-2 or COVID-19; (3) assessed the behavior and/or policy of mask wearing; and 4) conducted in community/public settings such as healthcare settings, worksites, and schools. Studies were excluded if (1) they did not parse out data specific to the effect of mask wearing (behavior and/or policy) and subsequent SARS-CoV-2 transmission or COVID-19; or (2) they relied solely on statistical models to estimate the effects of mask wearing on transmission (i.e., no primary data were collected on mask-wearing behavior or transmission). The time span for the literature search included all years available in the databases up to June 2023.

This study followed the structure and recommendations for reporting a systematic literature review as outlined by the Preferred Reporting Items for Systematic Reviews and Meta-Analyses (PRISMA) guidelines [18]. Searches were uploaded to the Rayyan platform (Cambridge, MA, USA), which was also used to manage reviewer ratings and remove duplicate studies. Database searches and gray literature searches were conducted by two independent reviewers. After identifying articles from each database, duplicates were identified and removed before screening for inclusion criteria. A total of 2616 studies were screened and evaluated first by title and abstract for meeting inclusion and exclusion criteria by a primary and secondary reviewer. Discrepancies between reviewers were resolved after a discussion following the initial screening. Articles that met criteria in phase 1 (n = 470) underwent full-text review (phase 2) by two independent reviewers. Final inclusion of articles was determined after the full-text review. The included articles were also explored for works cited by using the backward referencing technique to search for additional literature that met the inclusion criteria.

### 2.3. Data Extraction

For all articles that met the inclusion and exclusion criteria (n = 79), relevant data and study characteristics were extracted by the two independent reviewers following a standardized data abstraction protocol. These included author, year, country, study design, outcome measures, study sample, main finding, adherence, duration, and study limitations.

### 2.4. Risk of Bias Assessment and Outcome Analysis

Two independent reviewers assessed the quality of included studies using Joanna Briggs Institute (JBI) critical appraisal tools [19]. A third reviewer was consulted when discrepancies were identified. JBI critical appraisal checklists are considered suitable and the most preferred for scoring quasi-experimental, observational, and cross-sectional study designs, which were the majority of studies included in this review [20]. Checklists specific to study design were used to evaluate criteria such as description of clinical condition, validity of measurement of disease outcome, appropriateness of statistical approaches, and mitigation of confounders. Each JBI risk of bias criterion for each study was rated as either red = “no”, green = “yes”, or yellow = “unclear.” Risk of bias was ranked as high when the study was rated with ≤49% of “yes” scores, moderate when the study was rated between 50 and 79% of “yes” scores, and low when the study was rated with ≥80% of “yes” scores.

Due to the high heterogeneity of statistical methods and study designs in the included studies, a vote counting methodology was used in lieu of a meta-analysis to summarize the direction of effect for masking behavior and/or masking policies for the prevention of SARS-CoV-2 transmission and COVID-19 disease [21]. The primary objective of this approach was to compare studies indicating a ‘benefit’ (i.e., prevention of SARS-CoV-2 transmission or COVID-19) with those indicating a potential ‘harm’ (i.e., increased SARS-CoV-2 transmission or COVID-19). Two independent reviewers read through each study to identify the reported effect estimate as well as the observed direction of effect of a masking policy and/or mask wearing to develop a standardized binary metric. The number of effects demonstrating benefit was then compared to the number of effects indicating harm, allowing for a quantitative assessment of the overall effectiveness of mask-wearing behavior and/or policies. The certainty of evidence was graded by classifying SARS-CoV-2 transmission and COVID-19 results and rating the evidence for each outcome in the Gradepro GDT Standard version.

## 3. Results

**Figure 1:**
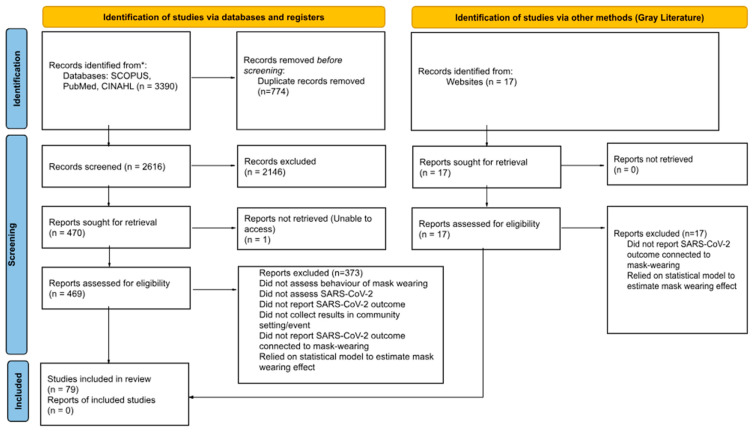
PRISMA diagram.

Results of the article screening process are presented in the PRISMA flow diagram (Figure 1). A total of 2616 articles and gray literature were identified, and 79 met the final criteria for inclusion and data abstraction. Table 2 summarizes key characteristics for each study included in this review. A greater proportion of studies (47%) were from the United States [22,23,24,25,26,27,28,29,30,31,32,33,34,35,36,37,38,39,40,41,42,43,44,45,46,47,48,49,50,51,52,53,54,55,56,57,58], followed by China (10%) [59,60,61,62,63,64,65,66], Germany (8%) [67,68,69,70,71,72], Japan (4%) [73,74,75], Spain (3%) [76,77], and other countries that had two or less studies (29%) [16,78,79,80,81,82,83,84,85,86,87,88,89,90,91,92,93,94,95,96,97,98,99]. SARS-CoV-2 infection was primarily identified through lab-verified test results such as polymerase chain reaction rapid (PCR) antigen tests (n = 56) or serological tests (n = 4), followed by self-report (n = 6) and aggregate community-level data reports of COVID-19 cases (n = 13).

### 3.1. Study Design and Vote Counting Results

Of the 79 studies, 34% (n = 27) were prospective or retrospective cohort studies, 29% (n = 23) were cross-sectional studies, 13% (n = 10) were case–control studies, 14% (n = 11) were case reports, 6% (n = 5) were quasi-experimental studies, and 4% (n = 3) were randomized controlled studies (RCTs). Overall, 77% (n = 61) of studies reported a beneficial association or effect of mask wearing or policy on SARS-CoV-2 transmission or COVID-19 disease (Table 3). Among the cross-sectional studies, 87% (n = 20) reported a beneficial association of mask wearing or policy on SARS-CoV-2 transmission or COVID-19. Ninety percent (n = 9) of case–control studies reported a beneficial effect of mask wearing or policy. Of the prospective cohort studies, 93% (n = 14) reported a benefit from mask wearing or policy, and of the retrospective cohort studies, 67% (n = 8) reported a benefit to mask wearing or policy. Lastly, all the quasi-experimental and RCT studies reported a benefit to mask wearing and preventing the spread of SARS-CoV-2 transmission or COVID-19 disease (n = 8). Most of the case report studies did not report sufficient information to determine whether mask wearing or policy provided any benefits or harm (82%, n = 9).

### 3.2. Type of Mask

There was high variability in the types of masks used in the studies that were evaluated (e.g., surgical, cloth, N-95, FFP2, and KF94 respirator). Of the total 79 included studies, 82% (n = 65) assessed various combinations of types of masks (e.g., two or more types). Among these studies, 77% (n = 50) found masks to be beneficial in preventing SARS-CoV-2 transmission or COVID-19 disease, 6% (n = 4) found no benefit, and 17% (n = 11) did not report sufficient data to determine the effect (Table 3). The remaining 18% (n = 14) of studies assessed either surgical masks (n = 10), N-95s (n = 3), or FFP2s (n = 1). Of the 10 studies that assessed surgical masks, 80% (n = 8) found this type of mask to be beneficial, one found no benefit, and one did not provide sufficient data to determine the effect (Table 3). All studies assessing N-95 masks (n = 3) and the single study on FFP2 masks reported them to be beneficial in reducing SARS-CoV-2 transmission.

### 3.3. Settings

#### 3.3.1. Community

A greater proportion of the studies occurred in community settings (n = 34, 43%) (Table 2). Community settings included studies conducted in neighborhood settings (n = 23), aircraft (n = 3), work offices/businesses (n = 2), nightclubs/music festivals (n = 2), sports facilities (n = 1), hair salons (n = 1), community markets (n = 1), or camps (n = 1). Among the 34 community studies, 91% (n = 31) found face masks to be beneficial in preventing SARS-CoV-2 transmission, 6% (n = 2) found no benefit, and 3% (n = 1) did not report sufficient data to determine an association. Of these studies, 28 (82%) examined various types of masks or did not specify the mask type; within this group, 25 (89%) found that mask use reduced transmission. Six studies (18%) specifically assessed surgical, N-95, FFP2, or two-layer cloth masks, and five (83%) demonstrated effectiveness in preventing transmission.

Four different outcome measures were used to assess SARS-CoV-2 infection and COVID-19 in community and neighborhood settings. Symptomatic laboratory-confirmed COVID-19 was the most common method used in 23 (68%) studies, with masks shown to be effective in 21 (91%) of them. Aggregate community transmission was assessed in seven studies (20%), in which five (71%) found masks to be protective. Two studies (6%) relied on participants’ self-reported laboratory-confirmed infection, and all reported a protective effect of mask use. Finally, SARS-CoV-2 seroconversion was evaluated in two studies (6%), with both showing benefit.

#### 3.3.2. Healthcare

A total of 30 studies (38%) took place in a healthcare setting (Table 2). Healthcare settings included public hospitals (n = 23), healthcare networks (n = 2), dental settings (n = 1), residential aged-care facilities (i.e., nursing home) (n = 1), tertiary care medical centers (n = 1), public health centers (n = 1), and Veteran’s Affairs (VA) healthcare centers (n = 1). Among these studies, 70% (n = 21) found facemasks or mask policies to be beneficial in preventing SARS-CoV-2 transmission or COVID-19 disease, whereas one found there to be no benefit. Additionally, 27% (n = 8) of studies did not report sufficient data to determine the association between face mask wearing and COVID-19. Of these studies, 16 (53%) assessed masks that included surgical, N-95, FFP2, FFP2, disposable, and KF94 respirators, and 10 (63%) of these studies reported the masks to prevent the transmission of SARS-CoV-2. Fourteen studies examined various masks or did not specify what kinds of masks were assessed. Twelve (86%) of the various or not specified masks in a community setting helped reduce the transmission of SARS-CoV-2.

Three outcome measures were used to assess SARS-CoV-2 infection and COVID-19 in healthcare settings. The most common was symptomatic laboratory-confirmed COVID-19, reported in 26 studies (74%), with masks shown to be effective in 17 (65%) of them. Two studies (7%) relied on participants’ self-reported laboratory-confirmed infection, and all reported a protective effect of mask use. Finally, SARS-CoV-2 seroconversion was evaluated in two studies (7%), with both showing benefit.

#### 3.3.3. Schools/Universities

Few studies took place in school settings (n = 15; 19%) (Table 2), which included K-12 schools (n = 12), classrooms (n = 1), universities (n = 1), and childcare programs (n = 1). Among these studies, 80% (n = 12) found facemasks to be beneficial in preventing SARS-CoV-2 transmission or COVID-19, 13% (n = 2) found no benefit, and 7% (n = 1) did not report sufficient data to determine an association. Of these studies, 14 (93%) examined various types of masks or did not specify the mask type; within this group, 10 (71%) found that masks reduced transmission. One study (7%) specifically assessed FFP2 masks and found that they demonstrated effectiveness in preventing transmission.

Three different outcome measures were used to assess SARS-CoV-2 and COVID-19 in school and university settings. Symptomatic laboratory-confirmed COVID-19 was used in seven (47%) studies, with masks shown to be effective in four (57%) of them. The second outcome measure was aggregate community transmission, which was used in six studies (40%), of which all found masks to be protective. Finally, two studies (13%) relied on participants’ self-reported laboratory-confirmed infection, and both reported a protective effect of mask use.

#### 3.3.4. Mask Mandates and Policies

Thirteen studies [25,28,29,30,31,32,34,37,42,49,55,70,94] specifically assessed the effectiveness of mask mandates and policies on COVID-19 outcomes (e.g., transmission rates, hospitalizations, and deaths) across statewide, county-level, and school settings. Overall, 85% (11 of 13) of studies demonstrated a beneficial effect in the prevention of COVID-19. Approximately 100 days after implementation, U.S. statewide mask mandates were reported to be associated with a 1.1% decrease in county-level COVID-19 cases [34]. In Kansas, counties that implemented mask mandates observed a decrease (mean decrease = 0.08 cases per 100,000 per day) in COVID-19 incidence, while those without mask mandates observed an increase (mean increase = 0.11 cases per 100,000 per day) [55]. Multiple studies in the U.S. and Germany found that mask mandates implemented in K-12 schools were associated with lower COVID-19 infection and transmission rates [31,37,49,70]. One observation study conducted across 61 U.S. school districts found that those with optional masking policies had a 3.6 times higher rate of secondary COVID-19 transmission [28]. However, one study did not find an association between the Texas Statewide Mandate (GA-29) and reductions in COVID-19 hospitalization rates or incidence [25]. Another study analyzed COVID-19 data across 35 European countries and found that countries that complied more with mask mandates did not have lower COVID-19 incidence rates [85].

### 3.4. Risk of Bias Assessment and Quality of Evidence

Table 4, Table 5, Table 6, Table 7, Table 8 and Table 9 show the risk of bias assessment across all studies included. Six separate JBI checklists were used to assess bias based on study design. Risk of bias was classified as high in 3 studies (3.8%), moderate in 31 studies (39.2%), and low in 45 studies (57%). Of the 11 case reports, 55% were classified as low risk of bias, while 45% were classified as moderate risk of bias due to insufficient description of adverse events (Table 4). Most (88%) of the quasi-experimental and RCT studies were classified as moderate or high bias due to unclear allocation procedures, lack of blinding, and the inadequate description of follow-up procedures to minimize dropout (Table 5 and Table 9). Of the case–control studies, 70% were rated as low risk of bias (Table 6). Fifty-six percent of cohort studies were classified as low risk of bias, and most were reliable in the measurement of outcomes, as they were laboratory-confirmed results (Table 7). Thirteen out of twenty-three (57%) cross-sectional studies were rated as low risk of bias (Table 8).

Table 10 summarizes the quality of evidence for four types of COVID-19 outcomes: (1) symptomatic laboratory-confirmed COVID-19, (2) self-reported laboratory-confirmed COVID-19, (3) SARS-CoV-2 seroconversion, and (4) aggregate community-level incidence (rt-qPCR). For all outcomes, the level of certainty for the evidence was low due to a high number of non-randomized studies and the number of studies with moderate to high risk of bias.

## 4. Discussion

Results of this systematic review demonstrate consistent evidence (77% of all studies), supporting that wearing a facemask and masking policies are effective at prevention and reducing the risk of SARS-CoV-2 transmission or COVID-19 disease across various real-world settings. Mask wearing was reported to reduce SARS-CoV-2 transmission across multiple community spaces (e.g., markets, workplaces, and salons), healthcare environments, and schools. These findings provide evidence that masking is an important and practical strategy to reduce transmission outside of controlled laboratory conditions. The results of this review are consistent with previous systematic reviews and meta-analyses [17,100,101,102]. One previous meta-analysis demonstrated a pooled relative risk of 0.12 (i.e., risk reduction of 88%), and another study reported an odds ratio of 0.38 for risk of SARS-CoV-2 infection when masking policies were in place [101,102]. Another review conducted only in cohort and case–control studies found that wearing a cloth mask decreased the odds of COVID-19 regardless of mask type [103]. The results of the present systematic review builds on these previous studies [17] and provides additional evidence for the benefits of face masks and masking policies to reduce SARS-CoV-2 transmission and COVID-19 disease specifically in real-world settings. This is particularly important given that the public’s adherence to wearing masks and the enforcement of mask wearing can vary widely, which would lower the expected effect of the policy. Yet, the results from this review show that 85% (11 of 13) of studies that focused on mask policies demonstrated a beneficial effect in preventing SARS-CoV-2 and COVID-19 disease. Thus, these findings support the external validity and generalizability of previous laboratory-based studies for the benefits of mask wearing to prevent SARS-CoV-2 infection and COVID-19 [100]. These results can be used to develop and support future public health messaging campaigns to address respiratory disease outbreaks, epidemics, and pandemics. Public health messaging that emphasizes positive framing for mask wearing such as promoting community togetherness and community unity resonate better with the general public compared to messages focused on fear or policies that may be perceived as overly intrusive [104].

This systematic review included studies of various designs, types of masks, and community settings to evaluate the evidence of effectiveness of mask wearing and masking policies. The proportion of studies demonstrating a benefit for mask wearing did not vary greatly by study design (67–100%), type of mask (77–100%), or setting (80–91%), while 85% of masking policies specifically reported a benefit. This level of consistent and protective effects of mask wearing and masking policies across study characteristics provides strong empirical evidence for the real-world effectiveness of masking recommendations to reduce the spread of SARS-CoV-2 and COVID-19 disease outside controlled settings. Importantly, the risk of bias ratings were classified as low for 57% of included studies. This suggests that the evidence from this systematic review can be interpreted as mostly reliable with moderate to high validity.

It is important to note that not all studies demonstrate a clear benefit of wearing face masks for the prevention of SARS-CoV-2 infection in community settings. In this systematic literature review, five studies (6%) did not demonstrate a clear protective effect. There are several potential explanations for these results. Sasser et al. [46] found no statistically significant association between COVID-19 incidence and face mask use among high school sports participants. This may be partially explained by a relatively low COVID-19 community prevalence during the time of the study. Likewise, April et al. [25] did not find that a mask mandate reduced SARS-CoV-2 transmission. It is also possible that mobility-restricting policies (i.e., stay-at-home orders) may have had a larger effect on reducing transmission than wearing masks in community settings. In these studies, data on self-reported mask wearing were not available, which may have limited the ability to evaluate the true effect of masking compared to other mitigation measures. Key study limitations included missing data and variable adherence to mask wearing, which can reduce the ability to detect an intervention effect. Notably, a previous Cochrane review concluded that N95 masks had little impact on the prevention of viral respiratory illness [105]. However, the studies included in the review demonstrated high variability in methodology and data quality, which can increase the risk of bias towards null findings [105]. These factors limit the ability to produce reliable estimates of effectiveness in community contexts. In summary, key methodological limitations among studies with null results may partially explain the non-significant findings for the effectiveness of mask wearing on the prevention of SARS-CoV-2 transmission and COVID-19 in community settings. Future research should address these methodological limitations to provide more accurate and reliable estimates of effectiveness.

### Strengths and Limitations

This review followed a rigorous and systematic approach using PRISMA guidelines to identify and evaluate relevant studies. Two independent reviewers followed a strict protocol for assessing inclusion/exclusion criteria and subsequently abstracting study data. The Rayyan study management platform and the JBI risk of bias appraisal tools provided standardized methods to manage and evaluate studies. However, a meta-analysis was not conducted due to the high heterogeneity of settings, types of masks, study designs, statistical methods, and outcome measures used (e.g., infection, symptomology) across studies. Meta-analyses are not recommended when there is this level of heterogeneity. Instead, the vote counting method is recommended as an alternative to meta-analysis, which allows for the identification of patterns (direction of results) across different studies. Several studies included in this review lacked information on the community’s adherence to wearing face masks, the specific the type of masks used, and there were inconsistencies in the type of incidence measure (SARS-CoV-2 vs. COVID-19 disease). These limitations make it difficult to more precisely evaluate the effectiveness of masking policies and mask wearing. Furthermore, only three randomized controlled study trials were identified. Although randomized controlled studies can provide stronger evidence of effectiveness and external validity, these study designs can also raise ethical concerns in the context of a new and deadly respiratory disease, such as COVID-19, where randomizing people or communities to not wear face masks may be considered unethical. Lastly, studies written or translated to a language other than English were not included. This may potentially bias results if the excluded studies reported different or contrary results to those studies that were included in this review.

## 5. Conclusions

Evidence from this systematic literature review supports that masking policies and mask wearing are beneficial to prevent and reduce the risk of SARS-CoV-2 infections and COVID-19 disease in various real-world settings. Importantly, relative to other recommendations (such as limiting travel, limiting group gatherings), wearing a face mask remains one of the most feasible, accessible, and effective non-pharmacologic public health interventions to prevent the spread of SARS-CoV-2. Given the varying adherence to masking from the public, there is a need to improve public health messaging to address misinformation and barriers to wearing masks. Reducing barriers to wearing masks can include providing high quality masks to those who otherwise do not have access to them or would not actively seek to obtain one on their own and by creating positive and supportive social environments that support mask wearing (i.e., depoliticizing and de-stigmatizing mask use).

## Figures and Tables

**Table 1:** List of key search terms.

“SARS-CoV-2”[MeSH Terms] OR “SARS-CoV-2”[All Fields] OR “COVID”[All Fields] OR “COVID-19”[MeSH Terms] OR “COVID-19”[All Fields]) AND (“masks”[MeSH Terms] OR “masks”[All Fields] OR “mask”[All Fields]) AND (“transmissibility”[All Fields] OR “transmissible”[All Fields] OR “transmissibilities”[All Fields] OR “transmissibility”[All Fields] OR “transmissible”[All Fields] OR “transmissibles”[All Fields] OR “transmission”[MeSH Subheading] OR “transmission”[All Fields] OR “transmissions”[All Fields]

**Table 2:** Included studies.

Author (Year)	Study Design	Country	Study Purpose	Study Sample	Setting	Mask Type	Measurement of COVID-19	Duration
Abaluck et al. (2022) [78]	Randomized Controlled Trial	Bangladesh	To conduct a cluster-randomized trial that handed out masks and conducted a range of mask-wearing promotional activities	Rural Bangladesh residents	Mosques, markets, the main entrance roads to villages, and tea stalls	Various	Seroprevalence; blood sample	November 2020–April 2021
Adawee et al. (2021) [22]	Cross Sectional	United States	To survey commonalities among HCW who tested positive for COVID-19 and to evaluate the effectiveness of the organizational intervention to require HCW to wear masks throughout their shift	HCW who tested positive for COVID-19 from the first positive test, which occurred 18 March 2020, to the last known positive test at the time of analysis, which occurred 6 May 2020 (n = 40), were included	Hospital	Not specified	Positive COVID-19 test; lab test	March–May 2020
Sertcelik (2023) [79]	Case–Control	Turkey	To evaluate the risk factors for COVID-19 in HCWs and the effectiveness of the measures taken for protection	HCWs; cases had a positive test, and each case matched with 3 controls who worked in the same unit at the time of the RT-PCR test of the case, had no symptoms, and tested negative	2 hospital buildings of the University in Ankara, Turkey	Various	Positive SARS-CoV-2 test; naso-oropharyngeal sample	March 2020–May 2020
Ambrosch et al. (2020) [67]	Quasi-Experimental	Germany	To investigate the extent to which the introduction of a strict hygiene bundle including a general mask requirement has an impact on the SARS-CoV-2 nosocomial rate in the pandemic environment	All inpatients from a maximum care hospital in Regensburg (Bavaria)	Hospital	Surgical	SARS-CoV-2 infection; lab test	March 2020–June 2020
Andrejko et al. (2021) [24]	Case–Control	United States	To address predictors of SARS-CoV-2 infection among participants who reported high-risk exposures, defined as social contact with an individual known or suspected to have been infected with SARS-CoV-2, within 2 weeks preceding participants’ SARS-CoV-2 tests	California residents	NA	Various	Positive and negative SARS-CoV-2 test; exposures assessed by interviews	February 2021–November 2021
Andrejko et al. (2022) [23]	Case–Control	United States	Face mask or respirator use was assessed among individuals who self-reported being in indoor public settings during the 2 weeks preceding testing and who reported no known contact with anyone with confirmed or suspected SARS-CoV-2 infection during this time	Persons who received a positive (case participants) or negative (control participants) SARS-CoV-2 test result	Public settings	Various	Positive (case participants) or negative (control participants) SARS-CoV-2 test result	18 February–1 December 2021
April et al. (2022) [25]	Retrospective Cohort in the Context of a Natural Experiment	United States	To compare COVID-19 case load, hospital bed use, and deaths before and after implementation of Texas Executive Order GA-29 mask order	Residents of Texas	Texas	Various	COVID-19 incidence cases	Pre-order period was from 19 June to 2 July 2020; post-order period was 17 July to 17 September 2020
Badri et al. (2021) [26]	Cross Sectional	United States	To identify behaviors and evaluate trends in COVID-19-mitigating practices in a predominantly Black and Hispanic population and to identify differences in practices by self-reported ethnicity	Random sample of adults who underwent SARS-CoV-2 testing at a safety-net healthcare system	Chicago, IL	Various	Laboratory confirmed SARS-CoV-2	April 2020–May 2020
Baker et al. (2022) [27]	Retrospective Cohort	United States	To investigate the effectiveness of prevention strategies in household settings, CDC partnered with four U.S. jurisdictions to describe Omicron household transmission during November 2021–February 2022	Persons with sequence-confirmed Omicron infection and their household contacts	Chicago IL, Milwaukee WI, Utah, Connecticut	Various	COVID-19 positive test; interview	November 2021–February 2022
Baumkötter et al. (2022) [68]	Prospective Cohort	Germany	Protective behavior and SARS-CoV-2 infection risk in the population—results from the Gutenberg COVID-19 study	Random individuals drawn by the regional registration offices	Germany	Various	RT-qPCR and two antibody immunoassays; self-reported COVID-19 test results were additionally considered	January 2020–June 2021
Boutzoukas et al. (2022) [28]	Prospective Cohort in the Context of a Natural Experiment	United States	To evaluate school masking policies and secondary SARS-CoV-2 transmission	Students and staff	United States Schools	Various	Data were provided as aggregate counts at the school level and were analyzed at an aggregate district level; therefore, details on cases including students versus staff were not available	July 2021–December 2021
Brandt et al. (2021) [69]	Case Report	Germany	Present data on the effectiveness of preventive measures against SARS-CoV-2 during an acute viral spread amongst healthcare professionals and aimed to retrace the dissemination of SARS-CoV-2	Prospectively recorded data of all employees in our department with symptoms of possible SARS-CoV-2 infection	Hospital	Surgical, FFP2	SARS-CoV-2 infection; rt-qPCR	March 2020–April 2020
Bruckhaus et al. (2022) [29]	Cross Sectional	United States	To discover the implications of government-enforced health policies for reopening public businesses amidst the pandemic and its effect on county-level infection rates	Eighty-three US counties (n = 83) that reported at least 20 000 cases	Public businesses	Various	Laboratory confirmed SARS-CoV-2	November 2020
Budzyn et al. (2021) [30]	Cross Sectional	United States	To assess the impact of masking in schools on COVID-19 incidence among K–12 students across the United States	520 US Counties	Schools	Various	COVID-19 cases; CDC COVID-19 Data Tracker	July 2021–September 2021
Bundgaard et al. (2021) [16]	RCT	Denmark	To assess whether recommending surgical mask use outside the home reduces wearers’ risk for SARS-CoV-2 infection in a setting where masks were uncommon and not among recommended public health measures	Adults spending more than 3 h per day outside the home without occupational mask use	Community-based	Surgical	SARS-CoV-2 infection; positive test	April 2020–June 2020
Chano et al. (2022) [73]	Cross Sectional	Japan	To evaluate the correlation between seroprevalence of SARS-CoV-2 antibodies among HCWs and the implementation of PPE and IPC	HCWs	Nine public hospitals designated for COVID-19 in Shiga Prefecture	N-95	Serological surveillance of SARS-CoV-2 antibodies and self-answered questionnaire	February 2021–November 2021
Chen et al. (2020) [59]	Case Report	China	To evaluate the seroprevalence of SARS-CoV-2 in a cohort of 105 HCWs exposed to COVID-19 patients using both EIA and microneutralization assay	105 HCWs exposed to four patients who were laboratory confirmed with COVID-19	Hospital	Disposable non-surgical face mask, surgical mask, or N-95 respiratory if wearing face mask	COVID-19 infection; RT-qPCR	January 2020–February 2020
Cheng et al. (2020) [60]	Cross Sectional	China	To assess the effect of community-wide mask usage to control COVID-19 in HKSAR	HKSAR of China	Community-based	Various	COVID-19 positivity; lab test	December 2019 to April 8 2020
Collatuzzo et al. (2022) [80]	Cross Sectional	Italy	Effectiveness of prevention of SARS-CoV-2 transmission among unvaccinated Italian healthcare workers	HCWs	Hospitals	Various	Rhino-pharyngeal swabs to detect SARS-CoV-2 RNA by RT-PCR in a reference laboratory, and databases were established to monitor and follow subjects; SARS-CoV-2 RNA was studied by a molecular test, AptimaTM SARS-CoV-2 Assay with the PantherTM Fusion System	March 2020–September 2020
Coma et al. (2022) [81]	Retrospective Cohort	Spain	Unraveling the role of the mandatory use of face covering masks for the control of SARS-CoV-2 in schools: a quasi-experimental study nested in a population-based cohort in Catalonia (Spain)	Children aged 3–11 years attending preschool (3–5 years, without FCM mandate) and primary education (6–11 years, with FCM mandate)	Schools in Catalonia (Spain)	Various	Incidence of SARS-CoV-2, SARs, and effective R*	September 2021–December 2021
Donovan et al. (2022) [31]	Prospective Cohort	United States	SARS-CoV-2 incidence in K–12 school districts with mask-required versus mask-optional policies—Arkansas, August–October 2021	Students and staff within school districts	School districts	Various	Self-report	August 2021–October 2021
Dorr et al. (2022) [82]	Prospective Cohort	Switzerland	Analyzed the SARS-CoV-2 risk for HCWs depending on cumulative exposure to patients with COVID-19 and assessed whether this risk can be modulated by the use of respirators compared with surgical masks	2919 volunteer HCWs	7 healthcare networks in Northern and Eastern Switzerland	Various	SARS-CoV-2-self-reported positive nasopharyngeal swab and/or antinucleocapsid seroconversion from baseline; self-reported mask type when come in contact 12 months later	September 2020–2021
Doung-Ngern et al. (2020) [83]	Case–Control	Thailand	To evaluate the effectiveness of mask wearing, handwashing, social distancing, and other personal protective measures against SARS-CoV-2 infection in public in Thailand	Randomized individuals throughout Thailand through contact tracing	Night clubs, boxing stadiums, and a state enterprise office in Thailand	Various	SARS-CoV-2 infection; RT-PCR	March–May 2020
Fischer et al. (2021) [32]	Cross Sectional	United States	To examine mask wearing policy and adherence in association with COVID-19 case rates	All 50 states and D.C.	All 50 states and D.C.	All	Mask wearing and physical distance policies, mask adherence, COVID-19 cases, and demographics	April–September 2020
Gettings et al. (2021) [33]	Prospective Cohort in the Context of a Natural Experiment	United States	To assess the impact of school-level prevention strategies on incidence of COVID-19 among students and staff before the availability of COVID-19 vaccines	169 K–5 schools	Schools	Not specified	Laboratory-confirmed reverse transcription–polymerase chain reaction or rapid antigen-positive test results self-reported to the school	November 2020–December 2020
Gras-Valentí et al. (2021) [76]	Prospective Cohort in the Context of a Natural Experiment	Spain	To evaluate the effectiveness of a program of control and prevention of COVID-19 in an academic general hospital in Spain	Patients with confirmed diagnosis of COVID-19	Alicante University General Hospital (AUGH)	Surgical mask	Number of COVID-19 cases and the type of contact that occurred in hospitalized patients and HCW	March 2020–April 2020
Guo et al. (2020) [61]	Case–Control	China	Aimed to study orthopedic surgeons, a particular group of HCWs not working on the front lines, as an indication of the overall infection situation of healthcare workers	Orthopedic surgeons and trainees who were infected with COVID-19 from 31 December 2019 to 24 February 2020 in the urban area of Wuhan	24 hospitals in the urban area of Wuhan	N-95 respirator and other masks	Survey to identify the orthopedic surgeons who were infected with COVID-19 in Wuhan; outcome: possible risk factors for COVID-19	December 2019–February 2020
Guy et al. (2021) [34]	Cross Sectional	United States	To examine the association of state-issued mask mandates and allowing on-premises restaurant dining with COVID-19 cases and deaths during March 1–December 31, 2020	Starting in April, 38 states and the District of Columbia (DC) issued mask mandates in 2020	38 states and DC	Various	Case growth rates; county-level data on state government websites	March 2020–December 2020
Hast et al. (2022) [35]	Cross Sectional	United States	Describes the prevalence of COVID-19 risk behaviors in an exposed population of students and school staff in the pre-vaccine era and identifies associations between these behaviors and testing positive for SARS-CoV-2	School staff and students	12 public school districts in Atlanta, GA	Various	COVID-19 test and risk behavior survey	December 2020–January 2021
Heinsohn et al. (2022) [70]	Retrospective Cohort	Germany	Infection and transmission risks of COVID-19 in schools and their contribution to population infections in Germany: a retrospective observational study using nationwide and regional health and education agency notification data	School staff and students	Schools	Various	SARS-CoV-2 school infections and transmission	March 2020–April 2022
Hendrix et al. (2020) [36]	Case Report	United States	To assess the role of source control in preventing COVID-19 transmission	139 clients exposed to two symptomatic hair stylists with confirmed COVID-19 while both the stylists and the clients wore face masks	Hair salon	Surgical and N-95	COVID-19 diagnosis; lab-confirmed COVID-19	May 2020
Hong et al. (2020) [62]	Retrospective Cohort	China	To describe the epidemiological trajectory and clinical features of these patients, and a cluster of 21 sequential local COVID-19 patients originated from a couple back from Wuhan among 57 close-contact individuals was detailed	127 patients, 71 male and 56 female confirmed to be infected with SARS-CoV-2	Hospital	Not specified	COVID-19 diagnosis; lab-confirmed COVID-19	January 2020–March 2020
Jarnig et al. (2022) [84]	Retrospective cohort	Austria	Test the effectiveness of mask wearing in a classroom setting	School students	Classroom	FFP2	SARS-CoV-2 detected by PCR tests	September 2021–April 2022
Jehn et al. (2021) [37]	Cross Sectional	United States	To evaluate the association between school mask policies and school-associated COVID-19 outbreaks in K–12 public non-charter schools open for in-person learning in Maricopa and Pima counties	K–12 public non-charter schools open for in-person learning in Maricopa and Pima counties	Schools	Not specified	School-associated outbreak; 2 or more lab-confirmed COVID-19 cases	July 2021–August 2021
Kim et al. (2021) [85]	Case Report	South Korea	To report exposure of HCWs during dental procedures on a mild symptomatic COVID-19 patient	A total of 48 persons were identified as exposed, including 15 HCWs at a dental clinic at Konkuk University Medical Center	Dental setting	Surgical, KF94 respirator	SARS-CoV-2 infection; rRT-PCR testing	May 2020
Klompas et al. (2021) [38]	Case Report	United States	To describe 3 cases of SARS-CoV-2 transmission with homologous whole-genome sequencing that occurred despite the use of medical masks and eye protection	All patients and employees newly diagnosed with SARS-CoV-2 at Brigham and Women’s Hospital in Boston	Hospital	Not specified	SARS-CoV-2 infection; lab test	November 2020–January 2021
Lio et al. (2021) [63]	Case–Control	China	To clarify the efficacy of these measures, and the results may provide valuable guidance to policymakers to educate the general public about how to reduce the individual-level risk of COVID-19 infection	Patients from Centro Hospitalar Conde de São Januário (C.H.C.S.J.)	Hospital and high-risk countries	Not specified	Laboratory-confirmed COVID-19	March–April 2020
Liu et al. (2021) [39]	Prospective Cohort	United States	To better understand the risk of SARS-CoV-2 transmission from a pediatric primary index case to household contacts living in Los Angeles County	Households met eligibility criteria if the index case was less than 18 years of age, reported a positive SARS-CoV-2 test, was the first member of their household with known lab-confirmed COVID-19 infection in the last 14 days, and resided in Los Angeles County	Households	Not specified	SARS-CoV-2 pediatric index cases; lab-confirmed SARS-CoV-2	December 2020–February 2021
Malik (2020) [86]	Case Report	Pakistan	To understand the efficacy and benefits of different types of respiratory protective equipment used by HCWs during the management of patients infected with the coronavirus	55-year-old woman with a history of diabetes mellitus who was positive for SARS-CoV-2	Intensive care unit—hospital	N-95 and surgical	COVID-19 infection; PCR	March 2020
Martin-Sanchez et al. (2021) [64]	Cross Sectional	China	To assess the settings where COVID-19 transmission occurred and determine the fraction of transmission events that occurred in settings where masks are not usually worn	Hong Kong Department of Health on local COVID-19 cases diagnosed up to 30 September 2020	Hong Kong	Various	COVID-19 transmission; lab test	January 2020–September 2020
Meylan et al. (2021) [87]	Cross Sectional	Switzerland	To assess the SARS-CoV-2 transmission in HCWs using seroprevalence as a surrogate marker of infection in our tertiary care center according to exposure	1874 participants at the tertiary care university center in Lausanne, Switzerland	Hospital	Surgical	COVID-19 infection; PCR	May 2020–June 2020
Moorthy et al. (2022) [40]	Quasi-Experimental (two interventions, no comparison group)	United States	Masking adherence in K–12 schools and SARS-CoV-2 secondary transmission	2 K-12 schools districts including students and staff (2400 students in district 1; 20,000 students in district 2)	Schools	Various	Monitoring mask adherence and transmission rates	April 2021–May 2021
Murray et al. (2022) [41]	Prospective Cohort	United States	To assess the association between masking children 2 years and older and subsequent childcare closure because of COVID-19	6654 childcare professionals	United States and United States Territories’ childcare programs	Various	Self-reported child masking	22 May to 8 June 2020 (baseline) and 26 May–23 June 2021 (follow-up)
Nir-Paz et al. (2020) [88]	Case report	Israel	To assess risk of transmission of SARS-CoV-2 during flights	11 citizens from the Diamond Princesses cruise ship in Japan	Commercial aircraft	FFP2 and surgical	COVID-19 positivity; RT-PCR	2 weeks (flight + isolation)
Pan et al. (2021) [65]	Retrospective Cohort	China	To describe the use of masks among HCW exposed to index cases of COVID-19 and to evaluate any association with infection rate	Healthcare workers at Zhongnan Hospital of Wuhan University	Hospital	Surgical	Self-reported use of surgical masks and gloves and were tested for severe acute respiratory syndrome coronavirus 2	December 2019–February 2020
Pauser et al. (2021) [71]	Case Report (data analyzed cross sectionally)	Germany	To analyze SARS-CoV-2 transmission during a professional sports event (2nd division professional basketball in Germany)	69 players, coaches, and other persons present at the sporting event in Germany	Indoor sports facility	Various	COVID-19 transmission; PCR test	November 2020
Ranjan et al. (2020) [89]	Cross Sectional	India	To determine whether the non-compliance with specific preventive practices was associated with the acquisition of the infection or not	384 patients of an outpatient COVID-19 clinic at a tertiary care hospital in New Delhi, India	Hospital	N-95	COVID-19 positivity; self-report preventative practices	June-July 2020
Rebeiro et al. (2021) [42]	Quasi-experimental	United States	To assess the impact of state mask-wearing requirement on COVID-19 cases, hospitalizations, and deaths in the United States	US residents	United States	Various	COVID-19 cases daily from 1 January 2020 to 31 October 2020	January 2020–October 2020
Rebmann et al. (2021) [43]	Cross Sectional	United States	To assess the impact of a modified quarantine protocol that considered mask use when determining which close contacts required quarantine	265 SLU students who received a positive SARS-CoV-2 test result	St. Louis University (SLU)	Not specified	Positive SARS-CoV-2 test; mask use	January 2021–May 2021
Reyné et al. (2021) [90]	Retrospective Cohort	France	The goal of this study was to investigate the efficiency of IPC measures implemented in the Hérault department (Occitanie region, France) in reducing the spread of SARS-CoV-2 in ACFs when a patient tested positive	Residents of the ACFs	12 public and private ACFs	Various	RT–PCR testing via nasopharyngeal swabs on a weekly basis; a full week without any residents testing positive for SARS-CoV-2 indicated the end of the outbreak	March 2020–May 2020
Riley et al. (2022) [44]	Prospective Cohort	United States	To examine how effective masks are at reducing transmission of SARS-CoV-2	Residents of Johnson County, Iowa who tested positive for COVID-19	Community	2-layer cloth masks, disposable surgical masks, double-layer gaiters, and KN-95 masks	Transmission of SARS-Co-2; lab-confirmed case of COVID-19	October 2020- February 2021
Russell et al. (2022) [91]	Quasi-Experimental **	Brazil	To estimate the individual effects of seven nonpharmaceutical interventions on COVID-19 cases and deaths to help policymakers choose the most effective interventions to mitigate the pandemic and reduce disease burden	COVID-19 cases and deaths who lived in Brazil	States of Brazil	Various	Case confirmation by PCR test	March 2020–December 2020
Sarti et al. (2021) [45]	Case Report	United States	To describe a COVID-19 cluster among workers in an office in Italy	Office workers	Office	Various	Nasopharyngeal swab results, symptoms onset, type of symptoms, and number and status of family members in respect to COVID-19	November 2020–December 2020
Sasser et al. (2022) [46]	Cross Sectional	United States	To describe the incidence of COVID-19 in Wisconsin high school athletes and investigate the relationship of COVID-19 incidence with sport and face mask use	Athletic directors representing 30,074 high school athletes with or without SARS-CoV2	High schools	Not specified	COVID-19 rates among athletes	September 2020
Seidelman et al. (2020) [47]	Prospective Cohort	United States	To measure the effect of universal masking on COVID-19 acquisition within the healthcare setting	From 15 March 2020 to 6 June 2020, all HCWs who tested positive for SARS-CoV-2 at Duke Health	Hospital	Not specified	COVID-19 incidence; negative binomial regression	March-June 2020
Shah et al. (2021) [48]	Retrospective Cohort	United States	Aimed to identify factors related to lapses in PPE use that may influence transmission of SARS-CoV-2 from patients to HCW	345 HCW who sustained a significant occupational exposure to a patient with COVID-19 from 13 May 2020 through 30 November 2020	Tertiary-care medical center in Minnesota	Surgical mask	COVID-19 evaluated by RT-PCR	May 2020–November 2020
Shah et al. (2022) [49]	Retrospective Cohort	United States	Data was collected from Texas school districts comparing COVID-19 positivity rates for districts where masks were mandated or optional	Students and staff from 30 school districts in Texas	Schools	Various	Positive COVID-19 test; lab test	August 2021–November 2021
Sharif et al. (2021) [92]	Cross Sectional	Bangladesh	This study was conducted to investigate the association of the preventive measures with the reduction in transmission of COVID-19 in Bangladesh	1690 participants from 54 districts in Bangladesh	Eight divisional cities covering 54 districts in Bangladesh	Various	Interviews over phone calls and by using digital questionnaires	January 2020–May 2021
Shaweno et al. (2021) [93]	Cross Sectional	Ethiopia	To determine the seroprevalence of SARS-CoV-2 antibody among individuals aged above 15 years and residing in the congregate settings of Dire Dawa city administration, Ethiopia	Individuals aged above 15 years and residing in randomly selected households from purposefully selected 11 enumeration areas in overcrowded neighborhoods in Dire Dawa	Overcrowded neighborhoods	Various	Seroprevalence, interview, and blood sample collection	June-July 2020
Spira (2022) [94]	Cross Sectional	European Countries	This analysis aimed to verify whether mask usage was correlated with COVID-19 morbidity and mortality	Individuals living in European countries	Western and Eastern European countries with a population of more than 1 million people	Various	Data on morbidity, mortality, and mask usage were retrieved from the IHME; vaccination obtained from Our World in Data	October 2020–March 2021
Sugimura et al. (2021) [74]	Cross Sectional	Japan	To investigate the relationship between mask wearing and COVID-19 among close contacts of COVID-19 patients	820 patients at public health centers in the Hiroshima Prefecture, Japan	Japanese public health centers	Various	Diagnosed COVID-19; PCR test	March–May 2020
Suh et al. (2021) [50]	Cross Sectional	United States	To estimate the prevalence of COVID-19 cases among campers and staff and its relation to individual and multiple NPIs instituted at these camps	US campers and staff	US ACA affiliated camps	Various	Surveys	Summer 2020
Sun et al. (2022) [95]	Prospective Cohort	Costa Rica	To better estimate the secondary attack rates and understand the behavioral determinants of SARS-CoV-2 household transmission, a household serologic study nested within a larger prospective population-based study of the SARS-CoV-2 immunologic response in Costa Rica was conducted	719 household contacts of 304 household index cases	Costa Rican households	Various	Blood specimens collected within 30–60 days of index case diagnosis, and serum was tested for presence of spike and nucleocapsid SARS-CoV-2 IgG antibodies	November 2020–July 2021
Suñer et al. (2022) [77]	Cross Sectional	Spain	The primary objective was to compare the incidence of COVID-19 within the 3 to 10 days following the event between attendees and a population-based control group	Attendees at a music festival	Two outdoor music festivals held in Catalonia	Various	Ag-RDT screening of nasopharyngeal swab for SARS-CoV-2 and survey-based assessment of risk behaviors during the event	3 July 2021 and 8–10 July 2021
Thakkar et al. (2022) [51]	Prospective Cohort	United States	Characterize school-associated secondary transmission and COVID-19 incidence among population of a single PreK-12 school during a period of high-community SARS-CoV-2 incidence	Students, faculty, and staff at a private school	Mecklenburg County, NC	Various	Self-report symptoms, community SARS-CoV-2 exposures, and any results of recent SARS-CoV-2 testing; cases defined by positive SARS-CoV-2 RT-PCR or antigen test result	August 2020–January 2021
Thompson et al. (2021) [52]	Retrospective Cohort	United States	To assess extent of a healthcare-associated outbreak of SARS-CoV-2 and to evaluate the effectiveness of infection control measures, including universal masking	Index patient and 250 exposed patients and staff	Integrated VA healthcare system with 2 facilities and 330 beds	Various	COVID-19 cases and transmission; point-prevalence survey	4 weeks
Tjaden et al. (2022) [53]	Case–Control	United States	To assess the association between self-reported mask wearing behavior during non-household interactions and COVID-19 infection during 3 pandemic periods using conditional logistic regression models of risk of infection that were adjusted for demographics, vaccination status, and recent known exposure to COVID-19	Sample of adults enrolled at 6 North Carolina healthcare systems	North Carolina healthcare systems	Various	Mask use, recent exposure, and positive SARS-CoV-2 test; self-report	April 2020–June 2021
Tjaden et al. (2023) [54]	Case–Control	United States	To assess the association between COVID-19 and consistent mask wearing during contact with others outside the household—a nested case–control analysis, November 2020–October 2021	Participants who were associated with 10 different healthcare systems	Southern United States	Various	Symptomatic SARS-CoV-2 infection (COVID-19)	November 2020–October 2021
Toyokawa et al. (2021) [75]	Prospective Cohort	Japan	Transmission of SARS-CoV-2 during a 2 h domestic flight to Okinawa, Japan, March 2020	146 aircraft passengers, excluding the pilots	Aircraft	Various	Measuring transmission of SARS-CoV-2 by nasopharyngeal swabs, PCR tests, and mask-wearing outcomes	23 March 2020
Van Dyke et al. (2020) [55]	Cross Sectional in the Context of a Natural Experiment	United States	To analyze trends in county-level COVID-19 incidence before (1 June–2 July) and after (3 July–23 August) the governor’s executive order among counties that ultimately had a mask mandate in place and those that did not	Mandated mask counties and nonmandated mask counties in Kansas	Community	Various	COVID-19 incidence; data from Kansas Health Institute	June 2020–August 2020
Varela (2022) [96]	RCT	Colombia	Aimed to determine the effectiveness of closed face shields with surgical face masks to prevent SARS-CoV-2 transmission in working adults during the COVID-19 pandemic in Bogotá, Colombia	CoVIDA project participants who had a negative RT-PCR test for SARS-CoV-2 in the previous 2 months	Participants living in geographic areas with active COVID-19 transmission and in areas with medium, medium-high, and high vulnerability index	Surgical mask	SARS-CoV-2 tested; lab test	January 2021–March 2021
Walker et al. (2020) [56]	Quasi-experimental in the Context of a Natural Experiment	United States	To describe the impact of universal masking and universal testing on admission for high-risk exposures to SARS-CoV-2 for HCWs	HCWs at a tertiary referral center in the Southeastern United States	Hospital	N-95	Universal masking; self-report of exposures to COVID-19	April 2020–May 2020
Wang et al. (2020) [66]	Retrospective Cohort	China	To study the use of NPIs, such as face masks, social distancing, and disinfection in the household setting	335 people in 124 families with at least one laboratory-confirmed COVID-19 case in Beijing	Households	N-95 mask, disposable surgical mask, or cloth mask	Secondary transmission of SARS-CoV-2 within the family	February 2020–March 2020
Wang et al. (2020) [57]	Prospective Cohort	United States	To describe SARS-CoV-2 PCR test positivity among HCWs before, during, and after implementation of a policy requiring universal masking of all HCWs and patients in a large healthcare system in Massachusetts	9850 tested HCWs at Mass General Brigham	Hospital	Surgical	SARS-CoV-2 infection; rRT-PCR testing	March–April 2020
Wendt et al. (2020) [72]	Case Report	Germany	To investigate potential transmissions of a symptomatic SARS-CoV-2-positive physician in a tertiary care hospital who worked for 15 cumulative hours without wearing a face mask	Patients/187 nurses and doctors/technical and medical assistants and other healthcare staff	Hospital	Not specified	Laboratory-confirmed COVID-19	March 2020
Williams et al. (2021) [58]	Prospective Cohort	United States	To assess the risk of SARS-CoV-2 transmission from universally masked HCWs to patients or residents	HCWs and patients	Hospitals	Various	Laboratory-confirmed COVID-19	October 2020–April 2021
Williamson et al. (2021) [97]	Case–Control	Australia	Transmission of SARS-CoV-2 Delta variant from an infected aircrew member on a short-haul domestic flight, Australia 2021	Flight passengers and crew members aboard the aircraft	Aircraft	Various	A survey for contact tracing and PCR tests was conducted on individuals aboard the flight	June 2021
Wilson et al. (2022) [98]	Case–Control *	France	To investigate socio-demographic factors and professional practice associated with the risk of COVID-19 among HCWs in health establishments	2058 respondents, respectively, 1363 (66.2%) and 695 (33.8%), in medical and medico-social establishments, including HCWs with and without contact with patients	Medical establishments and medico-social establishments in France	Surgical mask	SARS-CoV2 PCR or antigenic test	March 2021–June 2021
Xinias et al. (2021) [99]	Case Report	Greece	To report experience regarding a pediatric patient-case who had a COVID-19 infection, which was initially considered a common viral infection and was managed accordingly for the first 36 h while being hospitalized	Hippokration Hospital pediatric ward staff	Hospital	Surgical mask	COVID-19 transmission; PCR test	7–10 after exposure to infected patient

* Also cross sectional; ** intervention was a natural experiment. Note: HCW = healthcare workers; RT-PCR = Reverse Transcription Polymerase Chain Reaction; COVID-19 = Coronavirus Disease 2019; SARS-CoV-2 = severe acute respiratory syndrome coronavirus 2; CDC = Centers for Disease Control and Prevention; RT-qPCR = Reverse Transcription Quantitative Polymerase Chain Reaction; FFP2 = Filtering Facepiece Part 2; RCT = randomized controlled trial; PPE = personal protective equipment; IPC = Infection Prevention and Control; EIA = Enzyme Immunoassay; HKSAR = Hong Kong Special Administrative Region; RNA = Ribonucleic Acid; FCM = Face Covering Mask; SAR = Secondary Attack Rate; R* = Reproductive Number; AUGH = Alicante University General Hospital; SLU = St. Louis University; D.C. = District of Columbia; rRT-PCR = Real-time Reverse Transcription Polymerase Chain Reaction; ACF = aged-care facilities; NPI = Nonpharmaceutical Intervention; ACA = American Camp Association; IgG = Immunoglobulin G Antibodies; Ag-RDT = Antigen Rapid Diagnostic Test; VA = United States Department of Veterans Affairs; NA = Not Applicable; and IHME = Institute of Health Metrics and Evaluation.

**Table 3:** Vote counting method results.

Author (Year)	Measure of Effect Description	Effect
Abaluck et al. (2022) [78]	Prevalence ratios	1
Adawee et al. (2021) [22]	None	Unable to determine *
Ahmet Sertcelik (2023) [79]	Odds ratios	1
Ambrosch et al. (2020) [67]	Unadjusted incidence density ratio	1
Andrejko et al. (2021) [24]	Odds ratios	1
Andrejko et al. (2022) [23]	Odds ratios from multivariable logistic regression	1
April et al. (2022) [25]	Cohen’s d	0
Badri et al. (2021) [26]	Odds ratios from multivariable logistic regression	1
Baker et al. (2022) [27]	Attack rates	1
Baumkötter et al. (2022) [68]	Prevalence ratios	1
Boutzoukas et al. (2022) [28]	Incidence rate ratio	1
Brandt et al. (2021) [69]	None	Unable to determine *
Bruckhaus et al. (2022) [29]	Incidence rate comparison using multiple linear regression	1
Budzyn et al. (2021) [30]	Multiple linear regression	1
Bundgaard et al. (2021) [16]	Odds ratio from logistic regression	1
Chano et al. (2022) [73]	Odds ratios	1
Chen et al. (2020) [59]	Odds ratios from multivariable logistic regression	1
Cheng et al. (2020) [60]	Incidence rate comparison using exact Poisson test	1
Collatuzzo et al. (2022) [80]	Odds ratios from multivariable logistic regression	1
Coma et al. (2022) [81]	Risk ratios	0
Donovan et al. (2022) [31]	Incidence rate ratios	1
Dorr et al. (2022) [82]	Odds ratios	1
Doung-Ngern et al. (2020) [83]	Odds ratios from multivariable logistic regression	1
Fischer et al. (2021) [32]	Odds ratios from multivariable logistic regression	1
Gettings et al. (2021) [33]	Rate ratios from adjusted -1 binomial regression models	1
Gras-Valentí et al. (2021) [76]	Unadjusted relative risk ratios	1
Guo et al. (2020) [61]	Odds ratios from multivariable logistic regression	1
Guy et al. (2021) [34]	Weighted least-squares regression for COVID-19 case counts	1
Hast et al. (2022) [35]	Odds ratios	1
Heinsohn et al. (2022) [70]	Incidence rates	1
Hendrix et al. (2020) [36]	None; transmission did not occur among 67 close contacts	Unable to determine *
Hong et al. (2020) [62]	Comparison of incidence proportions	1
Jarnig et al. (2022) [84]	Odds ratio from logistic regression	1
Jehn et al. (2021) [37]	Odds ratios from multivariable logistic regression	1
Kim et al. (2021) [85]	None	Unable to determine *
Klompas et al. (2021) [38]	None	Unable to determine *
Lio et al. (2021) [63]	Odds ratios from multivariable logistic regression	1
Liu et al. (2021) [39]	Household secondary attack rates	1
Malik (2020) [86]	None	Unable to determine *
Martin-Sanchez et al. (2021) [64]	Odds ratios from multivariable logistic regression	1
Meylan et al. (2021) [87]	Odds ratios from multivariable logistic regression	1
Moorthy et al. (2022) [40]	Secondary transmission rates over time	1
Murray et al. (2022) [41]	Risk ratios	1
Nir-Paz et al. (2020) [88]	None	Unable to determine *
Pan et al. (2021) [65]	Descriptive comparison (*t*-test)	1
Pauser et al. (2021) [71]	Fisher’s exact test	1
Ranjan et al. (2020) [89]	Odds ratios from multivariable logistic regression	1
Rebeiro et al. (2021) [42]	Incidence rate slopes	1
Rebmann et al. (2021) [43]	Odds ratios from multivariable logistic regression	1
Reyné et al. (2021) [90]	Odds ratio from generalized linear mixed model	1
Riley et al. (2022) [44]	Odds ratios from multivariable logistic regression	1
Russell et al. (2022) [91]	Daily percent positivity growth rate	1
Sarti et al. (2021) [45]	None	Unable to determine *
Sasser et al. (2022) [46]	Incidence rate ratios from -1 binomial regression	0
Seidelman et al. (2020) [47]	Incidence rate comparison from -1 binomial regression	1
Shah et al. (2021) [48]	Student’s *t* test	0
Shah et al. (2022) [49]	Student’s *t* test comparing percent positivity	1
Sharif et al. (2021) [92]	Odds ratios	1
Shaweno et al. (2021) [93]	Odds ratios from multivariable logistic regression	1
Spira (2022) [94]	Correlation coefficient	0
Sugimura et al. (2021) [74]	Relative risk ratios from adjusted Poisson regression models	1
Suh et al. (2021) [50]	Risk ratios	1
Sun et al. (2022) [95]	Odds ratios	1
Suner et al. (2022) [77]	Odds ratios	1
Thakkar et al. (2022) [51]	None	Unable to determine *
Thompson et al. (2021) [52]	None	Unable to determine *
Tjaden et al. (2022) [53]	Odds ratios	1
Tjaden et al. (2023) [54]	Odds ratios from multivariable logistic regression	1
Toyokawa et al. (2021) [75]	Odds ratios from multivariable logistic regression	1
Van Dyke et al. (2020) [55]	Generalized estimating equation regression	1
Varela (2022) [96]	Risk difference	1
Walker et al. (2020) [56]	Rate ratios from unadjusted -1 binomial regression model	1
Wang et al. (2020) [66]	Odds ratios from multivariable logistic regression	1
Wang et al. (2020) [57]	Weighted non-linear regression of positivity rates	1
Wendt et al. (2020) [72]	None	Unable to determine *
Williams et al. (2021) [58]	Relative risk ratios	1
Williamson et al. (2021) [97]	None	Unable to determine *
Wilson et al. (2022) [98]	Odds ratios	1
Xinias et al. (2021) [99]	None	Unable to determine *

Note: 1 = benefit; 0 = no association; 1 = harm; and * study did not report sufficient data to determine if mask wearing or policy had an association with SARS-CoV-2 infection or COVID-19.

**Table 4:** JBI risk of bias quality assessment—case reports.

Study	A1	A2	A3	A4	A5	A6	A7	A8	Yes (%)	Risk *
Wendt et al. (2020) [72]	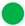	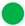	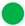	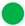	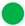	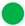	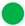	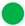	100	Low
Nir-Paz et al. (2020) [88]	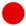	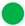	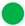	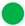	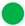	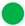	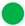	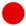	75	Moderate
Malik (2020) [86]	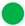	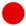	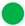	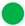	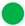	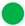	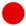	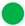	75	Moderate
Kim et al. (2021) [85]	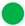	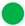	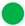	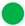	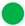	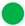	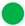	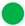	100	Low
Xinias et al. (2021) [99]	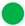	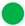	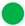	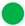	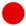	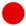	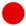	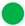	62.5	Moderate
Brandt et al. (2021) [69]	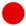	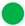	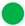	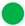	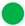	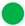	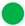	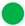	87.5	Low
Klompas et al. (2021) [38]	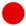	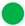	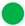	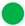	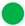	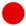	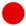	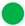	62.5	Moderate
Hendrix et al. (2020) [36]	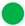	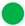	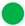	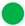	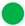	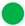	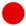	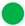	87.5	Low
Chen et al. (2020) [59]	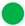	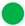	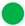	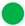	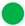	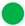	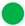	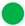	100	Low
Sarti et al. (2021) [45]	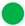	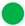	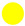	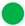	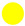	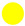	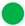	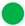	62.5	Moderate
Pauser et al. (2021) [71]	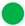	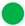	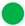	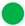	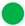	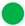	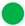	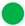	75	Low

Criteria Met: 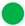 = yes; 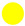 = unclear; and 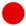 = no; A1 Were patients’ demographic characteristics clearly described?; A2 Was the patient’s history clearly described and presented as a timeline?; A3 Was the current clinical condition of the patient on presentation clearly described?; A4 Were diagnostic tests or assessment methods and the results clearly described?; A5 Was the intervention(s) or treatment procedure(s) clearly described?; A6 Was the post-intervention clinical condition clearly described?; A7 Were adverse events (harms) or unanticipated events identified and described?; A8 Does the case report provide takeaway lessons?; * Risk of bias was ranked as high when the study was rated with ≤49% of “yes” scores, moderate when the study was rated between 50 and 79% of “yes” scores, and low when the study was rated with ≥80% of “yes” scores.

**Table 5:** JBI risk of bias quality assessment—quasi-experimental trial.

Study	B1	B2	B3	B4	B5	B6	B7	B8	B9	Yes (%)	Risk *
Walker et al. (2020) [56]	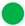	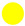	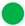	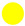	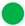	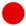	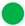	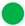	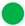	66.7	Moderate
Rebeiro, Aronoff, and Smith (2021) [42]	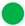	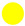	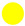	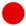	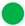	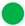	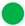	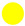	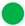	55.6	Moderate
Moorthy et al. (2022) [40]	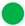	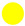	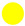	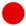	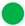	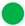	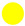	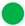	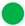	55.6	Moderate
Ambrosch et al. (2020) [67]	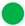	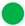	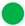	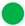	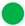	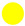	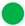	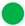	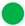	88.9	Low
Russell et al. (2022) [91]	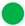	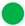	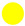	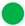	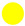	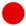	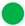	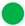	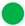	66.7	Moderate

Criteria Met: 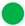 = yes; 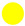 = unclear; and 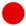 = no; A1 Were patients’ demographic characteristics clearly described?; A2 Was the patient’s history clearly described and presented as a timeline?; A3 Was the current clinical condition of the patient on presentation clearly described?; A4 Were diagnostic tests or assessment methods and the results clearly described?; A5 Was the intervention(s) or treatment procedure(s) clearly described?; A6 Was the post-intervention clinical condition clearly described?; A7 Were adverse events (harms) or unanticipated events identified and described?; A8 Does the case report provide takeaway lessons?; * Risk of bias was ranked as high when the study was rated with ≤49% of “yes” scores, moderate when the study was rated between 50 and 79% of “yes” scores, and low when the study was rated with ≥80% of “yes” scores.

**Table 6:** JBI risk of bias quality assessment—case–control studies.

Study	C1	C2	C3	C4	C5	C6	C7	C8	C9	C10	Yes (%)	Risk *
Lio et al. (2021) [63]	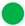	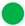	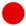	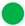	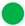	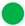	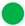	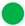	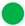	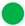	90	Low
Guo et al. (2020) [61]	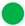	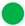	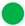	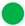	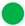	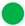	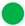	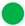	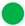	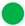	100	Low
Doung-Ngern et al. (2020) [83]	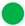	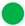	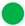	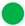	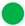	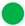	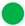	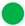	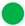	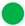	100	Low
Ahmet et al. (2023) [79]	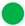	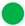	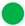	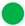	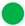	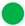	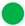	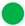	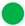	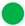	90	Low
Tjaden et al. (2022) [53]	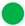	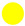	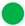	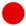	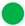	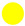	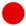	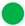	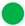	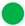	60	Moderate
Andrejko et al. (2022) [23]	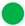	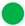	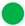	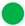	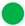	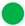	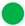	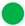	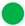	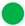	100	Low
Andrejko et al. (2021) [24]	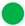	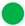	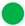	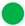	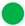	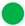	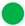	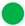	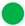	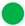	100	Low
Tjaden et al. (2023) [54]	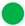	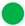	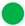	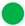	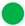	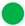	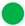	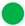	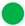	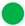	100	Low
Wilson et al. (2022) [98]	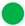	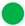	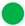	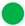	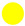	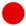	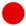	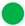	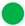	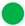	70	Moderate
Williamson et al. (2021) [97]	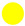	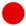	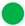	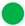	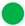	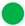	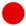	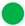	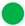	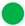	70	Moderate

Criteria Met: 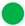 = yes; 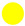 = unclear; and 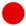 = no; A1 Were patients’ demographic characteristics clearly described?; A2 Was the patient’s history clearly described and presented as a timeline?; A3 Was the current clinical condition of the patient on presentation clearly described?; A4 Were diagnostic tests or assessment methods and the results clearly described?; A5 Was the intervention(s) or treatment procedure(s) clearly described?; A6 Was the post-intervention clinical condition clearly described?; A7 Were adverse events (harms) or unanticipated events identified and described?; A8 Does the case report provide takeaway lessons?; * Risk of bias was ranked as high when the study was rated with ≤49% of “yes” scores, moderate when the study was rated between 50 and 79% of “yes” scores, and low when the study was rated with ≥80% of “yes” scores.

**Table 7:** JBI risk of bias quality assessment—cohort studies.

Study	D1	D2	D3	D4	D5	D6	D7	D8	D9	D10	D11	Yes (%)	Risk *
Wang et al. (2020) [66]	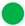	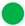	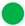	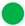	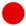	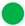	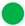	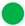	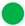	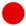	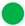	82	Low
Wang et al. (2020) [57]	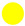	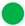	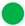	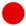	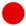	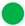	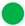	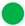	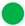	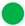	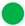	64	Moderate
Liu et al. (2021) [39]	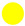	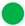	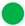	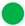	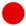	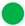	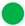	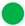	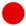	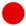	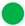	64	Moderate
Hong et al. (2020) [62]	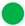	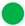	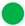	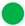	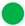	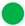	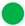	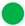	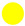	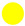	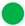	82	Low
Riley et al. (2022) [44]	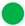	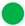	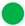	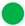	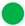	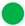	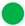	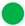	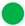	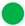	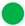	100	Low
Coma et al. (2022) [81]	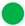	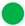	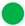	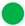	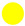	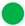	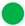	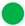	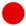	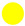	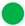	73	Moderate
Baumkötter et al. (2022) [68]	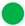	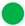	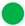	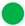	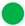	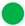	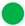	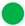	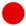	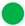	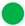	91	Low
Boutzoukas et al. (2022) [28]	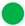	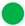	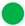	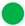	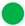	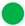	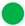	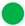	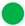	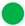	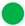	100	Low
Donovan et al. (2022) [31]	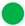	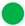	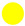	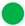	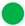	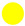	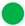	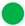	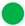	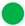	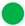	82	Low
Toyokawa et al. (2021) [75]	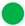	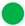	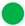	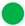	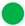	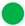	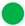	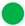	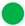	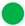	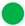	100	Low
April et al. (2022) [25]	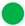	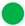	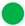	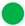	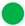	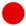	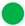	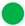	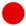	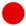	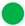	73	Moderate
Dörr et al. (2022) [82]	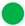	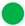	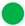	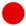	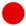	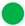	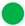	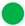	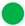	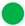	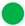	82	Low
Murray et al. (2022) [41]	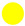	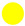	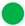	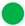	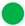	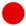	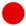	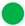	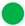	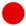	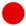	45	High
Seidelman et al. (2020) [47]	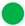	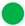	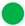	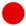	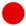	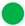	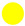	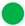	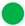	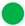	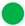	73	Moderate
Thakkar et al. (2022) [51]	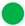	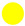	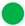	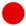	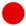	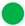	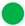	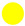	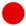	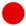	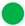	45	High
Williams et al. (2021) [58]	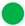	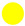	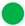	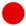	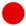	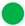	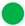	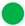	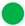	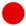	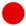	54.5	Moderate
Baker et al. (2022) [27]	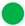	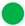	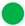	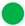	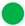	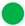	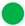	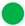	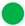	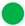	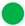	100	Low
Gras-Valentí et al. (2021) [76]	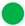	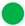	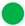	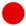	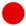	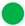	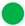	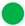	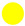	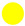	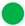	63	Moderate
Heinsohn et al. (2022) [70]	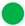	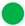	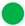	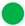	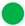	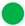	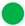	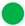	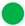	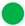	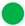	100	Low
Gettings et al. (2021) [33]	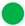	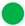	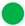	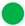	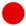	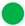	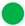	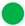	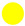	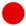	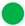	73	Moderate
Jarnig et al. (2022) [84]	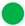	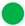	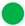	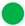	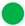	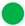	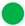	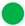	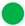	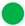	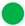	100	Low
Pan et al. (2021) [65]	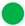	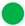	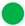	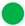	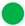	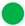	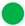	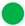	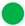	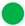	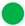	100	Low
Reyné et al. (2021) [90]	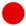	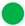	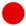	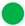	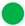	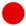	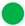	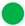	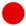	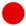	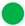	54.5	Moderate
Shah et al. (2021) [48]	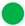	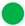	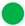	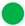	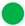	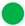	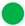	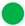	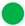	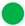	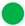	100	Low
Shah et al. (2022) [49]	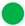	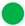	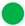	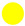	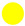	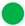	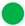	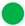	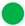	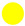	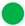	73	Moderate
Sun et al. (2022) [95]	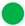	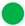	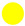	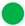	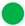	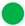	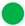	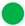	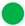	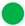	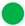	91	Low
Thompson et al. (2021) [52]	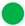	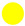	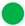	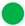	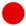	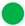	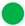	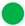	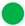	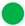	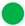	82	Low

Criteria Met: 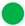 = yes; 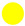 = unclear; and 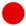 = no; A1 Were patients’ demographic characteristics clearly described?; A2 Was the patient’s history clearly described and presented as a timeline?; A3 Was the current clinical condition of the patient on presentation clearly described?; A4 Were diagnostic tests or assessment methods and the results clearly described?; A5 Was the intervention(s) or treatment procedure(s) clearly described?; A6 Was the post-intervention clinical condition clearly described?; A7 Were adverse events (harms) or unanticipated events identified and described?; A8 Does the case report provide takeaway lessons?; * Risk of bias was ranked as high when the study was rated with ≤49% of “yes” scores, moderate when the study was rated between 50 and 79% of “yes” scores, and low when the study was rated with ≥80% of “yes” scores.

**Table 8:** JBI risk of bias quality assessment—cross-sectional studies.

Study	E1	E2	E3	E4	E5	E6	E7	E8	Yes (%)	Risk *
Sugimura et al. (2021) [74]	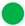	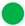	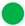	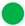	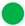	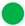	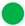	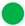	100	Low
Ranjan et al. (2020) [89]	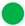	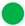	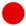	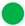	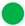	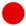	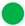	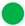	75	Moderate
Meylan et al. (2021) [87]	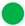	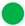	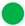	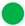	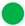	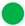	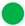	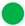	100	Low
Guy et al. (2021) [34]	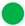	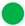	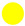	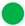	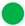	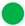	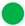	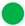	87.5	Low
Fischer et al. (2021) [32]	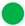	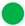	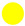	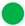	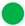	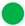	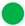	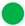	87.5	Low
Cheng et al. (2020) [60]	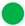	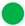	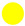	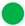	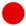	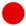	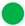	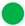	62.5	Moderate
Bruckhaus et al. (2022) [29]	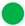	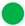	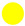	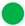	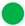	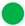	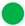	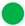	87.5	Low
Badri et al. (2021) [26]	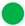	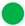	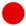	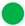	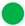	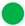	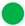	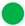	87.5	Low
Adawee et al. (2021) [22]	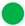	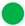	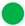	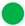	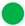	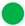	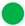	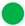	100	Low
Sasser et al. (2022) [46]	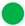	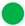	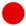	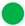	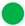	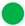	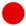	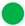	75	Moderate
Shaweno et al. (2021) [93]	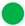	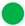	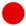	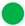	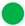	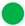	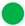	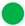	87.5	Low
Martin-Sanchez et al. (2021) [64]	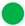	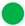	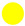	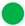	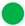	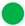	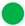	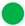	87.5	Low
Sharif et al. (2021) [92]	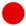	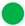	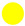	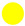	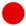	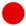	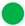	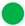	37.5	High
Spira (2022) [94]	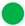	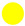	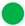	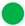	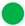	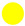	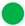	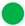	75	Moderate
Hast et al. (2022) [35]	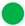	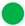	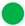	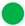	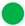	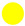	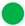	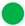	87.5	Low
Collatuzzo et al. (2022) [80]	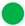	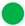	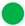	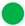	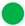	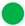	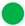	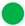	100	Low
Chano et al. (2022) [73]	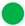	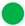	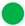	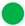	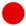	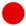	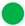	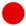	62.5	Moderate
Budzyn et al. (2021) [30]	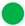	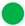	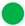	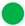	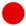	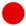	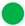	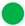	75	Moderate
Jehn et al. (2021) [37]	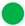	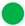	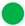	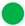	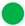	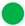	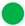	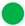	100	Low
Rebmann et al. (2021) [43]	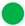	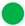	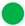	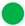	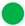	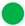	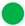	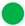	100	Low
Suh et al. (2021) [50]	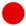	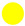	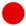	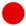	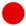	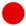	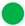	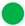	25	High
Suñer et al. (2022) [77]	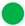	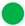	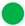	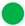	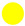	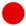	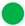	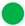	75	Moderate
Van Dyke et al. (2020) [55]	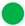	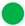	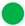	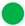	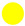	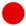	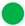	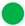	75	Moderate

Criteria Met: 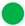 = yes; 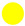 = unclear; and 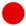 = no; A1 Were patients’ demographic characteristics clearly described?; A2 Was the patient’s history clearly described and presented as a timeline?; A3 Was the current clinical condition of the patient on presentation clearly described?; A4 Were diagnostic tests or assessment methods and the results clearly described?; A5 Was the intervention(s) or treatment procedure(s) clearly described?; A6 Was the post-intervention clinical condition clearly described?; A7 Were adverse events (harms) or unanticipated events identified and described?; A8 Does the case report provide takeaway lessons?; * Risk of bias was ranked as high when the study was rated with ≤49% of “yes” scores, moderate when the study was rated between 50 and 79% of “yes” scores, and low when the study was rated with ≥80% of “yes” scores.

**Table 9:** JBI risk of bias quality assessment—randomized controlled trial.

Study	F1	F2	F3	F4	F5	F6	F7	F8	F9	F10	F11	F12	F13	Yes (%)	Risk *
Bundgaard et al. (2021) [16]	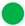	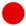	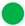	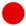	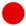	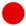	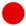	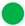	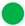	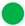	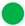	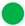	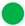	61.5	Moderate
Varela et al. (2022) [96]	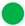	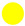	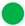	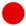	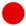	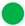	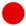	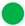	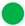	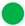	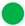	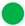	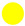	61.5	Moderate
Abaluck et al. (2022) [78]	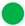	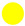	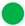	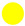	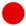	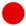	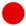	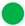	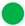	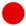	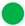	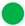	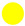	53.8	High

Criteria Met: 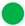 = yes; 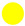 = unclear; and 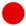 = no; A1 Were patients’ demographic characteristics clearly described?; A2 Was the patient’s history clearly described and presented as a timeline?; A3 Was the current clinical condition of the patient on presentation clearly described?; A4 Were diagnostic tests or assessment methods and the results clearly described?; A5 Was the intervention(s) or treatment procedure(s) clearly described?; A6 Was the post-intervention clinical condition clearly described?; A7 Were adverse events (harms) or unanticipated events identified and described?; A8 Does the case report provide takeaway lessons?; * Risk of bias was ranked as high when the study was rated with ≤49% of “yes” scores, moderate when the study was rated between 50 and 79% of “yes” scores, and low when the study was rated with ≥80% of “yes” scores.

**Table 10:** Summary of findings—face masks and masking policies for preventing SARS-CoV-2 transmission and COVID-19 disease.

Certainty Assessment							Description of Effect	Certainty	Importance
№ of Studies	Study Design	Risk of Bias	Inconsistency	Indirectness	Imprecision	Other Considerations	Masking Strategies and Policies		
**Symptomatic laboratory-confirmed COVID-19 (assessed with diagnostic lab test (rt-qPCR))**									
56	non-randomized studies	serious ^a^	serious ^b^	not serious ^b^	not serious	strong association, all plausible residual confounding would reduce the demonstrated effect	**Overall results:** Based on vote counting table, 39 out of the 56 (70%) included studies demonstrated a favorable effect of mask wearing in prevention of SARS-CoV-2 infection or COVID-19 disease. **Results by study design:** All 8 included case–control studies demonstrated a favorable association between mask wearing and COVID-19. However, 8 out of 11 case report studies did not report the effect between mask wearing and COVID-19. Fourteen cross-sectional studies were included, of which the majority (86%) reported a positive effect. Nineteen cohort studies were included, of which 14 reported a favorable effect between mask wearing and COVID-19. Four interventions (2 RCTs and 2 quasi-experimental) were included; all four studies reported a favorable effect between mask wearing and COVID-19 infection.	⨁⨁◯◯ Low ^a, b^	IMPORTANT
**Self-reported laboratory-confirmed COVID-19**									
6	non-randomized studies	serious ^c^	not serious	not serious	not serious	all plausible residual confounding would reduce the demonstrated effect	**Overall results:** Based on vote counting table, all 6 studies (100%) demonstrated a favorable effect of mask wearing in prevention of SARS-CoV-2 infection or COVID-19 disease. **Results by study design:** Of the six included studies, 2 cohort studies and 2 cross-sectional studies reported a favorable effect of mask wearing on COVID-19 infection. The 2 other studies included (case report and quasi-experimental) also demonstrated a favorable effect.	⨁⨁◯◯ Low ^c^	IMPORTANT
**SARS-CoV-2 seroconversion**									
4	non-randomized studies	serious ^d^	not serious	not serious	not serious	all plausible residual confounding would reduce the demonstrated effect	**Results by study design:** All four of the included studies (100%; 2 cross-sectional, a cohort, and a case–control study) demonstrated a favorable effect of mask wearing in prevention of SARS-CoV-2 infection or COVID-19 disease.	⨁⨁◯◯ Low ^d^	IMPORTANT
**Aggregate community-level incidence (rt-qPCR)**									
13	non-randomized studies	serious ^e^	not serious	not serious	not serious	all plausible residual confounding would reduce the demonstrated effect	**Overall results:** Eleven out of thirteen studies (85%) reported a favorable effect between mask wearing in prevention of SARS-CoV-2 infection or COVID-19 disease. **Results by study design:** All studies except 2 (1 cross-sectional and 1 retrospective cohort) demonstrated a favorable effect of mask wearing in prevention of SARS-CoV-2 infection or COVID-19 disease.	⨁⨁◯◯ Low ^e^	IMPORTANT

GRADE Working Group grades of evidence: Level of certainty is indicated by ⨁ selected; High certainty: It is highly improbable that additional research will alter confidence in the effect estimate. Moderate certainty: Additional research is likely to significantly influence confidence in the effect estimate and may lead to a change in the estimate. Low certainty: Additional research is highly likely to significantly affect confidence in the effect estimate and is expected to result in a change to the estimate. Very low certainty: There is a high degree of uncertainty regarding the estimate. Explanations: ^a^. We downgraded the quality of evidence for this outcome by 1 level, as several included studies had an overall moderate or high risk of bias (see JBI figures for risk of bias ratings). ^b^. Thirteen studies did not report an effect between mask wearing and COVID-19. Three studies reported no association between mask wearing and COVID-19. As we were not able to interpret these study findings and observed an inconsistent result in 3 studies, we decided to downgrade the evidence by one level. ^c^. Of the 6 included studies, 5 were rated as having moderate or high risk of bias (see JBI figures for risk of bias ratings). ^d^. Of the 4 included studies, 2 were rated as having moderate risk of bias (see JBI figures for risk of bias ratings). ^e^. Several studies were rated as having moderate risk of bias (see JBI figures for risk of bias ratings).

## Data Availability

All data from the current study are available from the corresponding author on reasonable request.
